# Information-based measure of disagreement for more than two observers: a useful tool to compare the degree of observer disagreement

**DOI:** 10.1186/1471-2288-13-47

**Published:** 2013-03-22

**Authors:** Teresa Henriques, Luis Antunes, João Bernardes, Mara Matias, Diogo Sato, Cristina Costa-Santos

**Affiliations:** 1 Health Information and Decision Sciences Department, Faculty of Medicine, University of Porto, Al. Prof. Hernâni Monteiro, Porto, 4200-319, Portugal; 2 Instituto de Telecomunicações, Porto, Portugal; 3 Centre for Research in Health Technologies and Information Systems (CINTESIS), Porto, Portugal; 4 Computer Science Department, Faculty of Science, University of Porto, Porto, Portugal; 5 Obstetrics and Gynecology Department, Faculty of Medicine, University of Porto, Porto, Portugal; 6 Instituto de Engenharia Biomédica, Porto, Portugal

## Abstract

**Background:**

Assessment of disagreement among multiple measurements for the same subject by different observers remains an important problem in medicine. Several measures have been applied to assess observer agreement. However, problems arise when comparing the degree of observer agreement among different methods, populations or circumstances.

**Methods:**

The recently introduced information-based measure of disagreement (IBMD) is a useful tool for comparing the degree of observer disagreement. Since the proposed IBMD assesses disagreement between two observers only, we generalized this measure to include more than two observers.

**Results:**

Two examples (one with real data and the other with hypothetical data) were employed to illustrate the utility of the proposed measure in comparing the degree of disagreement.

**Conclusion:**

The IBMD allows comparison of the disagreement in non-negative ratio scales across different populations and the generalization presents a solution to evaluate data with different number of observers for different cases, an important issue in real situations.

A website for online calculation of IBMD and respective 95% confidence interval was additionally developed. The website is widely available to mathematicians, epidemiologists and physicians to facilitate easy application of this statistical strategy to their own data.

## Background

As several measurements in clinical practice and epidemiologic research are based on observations made by health professionals, assessment of the degree of disagreement among multiple measurements for the same subjects under similar circumstances by different observers remains a significant problem in medicine. If the measurement error is assumed to be the same for every observer, independent of the magnitude of quantity, we can estimate within-subject variability for repeated measurements by the same subject with the within-subject standard deviation, and the increase in variability when different observers are applied using analysis of variance 1. However this strategy is not appropriate for comparing the degree of observer disagreement among different populations or various methods of measurement. Bland and Altman proposed a technique to compare the agreement between two methods of medical measurement allowing multiple observations per subject 2 and later Schluter proposed a Bayesian approach 3. However, problems arise when comparing the degree of observer disagreement between two different methods, populations or circumstances. For example, one issue is whether during visual analysis of cardiotocograms, observer disagreement in estimation of the fetal heart rate baseline in the first hour of labor is significantly different from that in the last hour of labor when different observers assess the printed one-hour cardiotocography tracings. Another issue that remains to be resolved is whether interobserver disagreement in head circumference assessment by neonatologists is less than that by nurses. To answer to this question, several neonatologists should evaluate the head circumference in the same newborns under similar circumstances, followed by calculation of the measure of interobserver agreement, and the same procedure repeated with different nurses. Subsequently, the two interobserver agreement measures should be compared to establish whether interobserver disagreement in head circumference assessment by neonatologists is less than that by nurses.

Occasionally, intraclass correlation coefficient (ICC), a measure of reliability, and not agreement 4 is frequently used to assess observer agreement in situations with multiple observers without knowing the differences between the numerous variations of the ICC 5. Even when the appropriate form is applied to assess observer agreement, the ICC is strongly influenced by variations in the trait within the population in which it is assessed 6. Consequently, comparison of ICC is not always possible across different populations. Moreover important inconsistencies can be found when ICC is used to assess agreement 7.

Lin’s concordance correlation coefficient (CCC) is additionally applicable to situations with multiple observers. The Pearson coefficient of correlation assesses the closeness of data to the line of best fit, modified by taking into account the distance of this line from the 45-degree line through the origin 8,9,10,11,12,13. Lin objected to the use of ICC as a way of assessing agreement between methods of measurement, and developed the CCC. However, similarities exist between certain specifications of the ICC and CCC measures. Nickerson, C. 14showed the asymptotic equivalence among the ICC and CCC estimators. However, Carrasco and Jover 15demonstrated the equivalence between the CCC and a specific ICC at parameter level. Moreover, a number of limitations of ICC, such as comparability of populations and its dependence on the covariance between observers, described above, are also present in CCC 16. Consequently, CCC and ICC to measure observer agreement from different populations are valid only when the measuring ranges are comparable 17.

The recently introduced information-based measure of disagreement (IBMD) provides a useful tool to compare the degree of observer disagreement among different methods, populations or circumstances 18. However, the proposed measure assesses disagreement only between two observers, which presents a significant limitation in observer agreement studies. This type of study generally requires more than just two observers, which constitutes a very small sample set.

Here, we have proposed generalization of the information-based measure of disagreement for more than two observers. As sometimes in real situations some observers do not examine all the cases (missing data), our generalized IBMD is set to allow different numbers of examiners for various observations.

## Methods

### IBMD among more than two observers

A novel measure of disagreement, denoted ‘information-based measure of disagreement’ (IBMD), was proposed 18 on the basis of Shannon’s notion of entropy 19, described as the average amount of information contained in a variable. In this context, the sum over all logarithms of possible outcomes of the variable is a valid measure of the amount of information, or uncertainty, contained in a variable 19. IBMD, use logarithms to measures the amount of information contained in the differences between two observations. This measure is normalized and satisfies the flowing properties: it is a metric, scaled invariant with differential weighting 18.

N was defined as the number of cases and x_ij_ as observation of the subject i by observer j. The disagreement between the observations made by observer pair 1 and 2 was defined as:

$$\mathrm{IBMD}=\frac{1}{\text{N}}\sum_{\text{i}=1}^{\text{N}}\log_{2}\left(\frac{\left|{\text{x}}_{\text{i}1}-{\text{x}}_{\text{i}2}\right|}{\max\left({\text{x}}_{\text{i}1},{\text{x}}_{\text{i}2}\right)}+1\right)$$

We aim to measure the disagreement among measurements obtained by several observers, allowing different number of observations in each case. Thus, maintaining ‘N’ as the number of cases, we consider M_i_, i = 1,..,N, as the number of observations in case i.

Therefore considering N vectors, one for each case, (x_11_,…,x_1*M*1_),…,(x_*N*1_,…,x_*NMN*_) with non-negative components, the generalized information-based measure of disagreement is defined as:

$$\mathrm{IBMD}=\frac{1}{\sum {}_{\text{i}=1}^{\text{N}}{\text{C}}_{2}^{M_{i}}}\sum {}_{\text{i}=1}^{\text{N}}\sum {}_{\text{j}=1}^{M_{i}-1}\sum {}_{\text{k}=\text{j}+1}^{M}\;\log\left(\frac{\left|{\text{x}}_{\text{ij}}-{\text{x}}_{\text{ik}}\right|}{\max\left({\text{x}}_{\text{ij}},{\text{x}}_{\text{ik}}\right)}+1\right)$$

with the convention $\frac{\left|0-0\right|}{\max\left(0,0\right)}=0$

This coefficient equals 0 when the observers agree or when there is no disagreement, and increases to 1 when the distance, i.e. disagreement among the observers, increases.

The standard error and confidence interval was based on the nonparametric bootstrap, by resampling the subjects/cases with replacement, in both original and generalized IBMD measures. The bootstrap uses the data from a single sample to simulate the results if new samples were repeated over and over. Bootstrap samples are created by sampling with replacement from the dataset. A good approximation of the 95% confidence interval can be obtained by computing the 2.5th and 97.5th percentiles of the bootstrap samples. Nonparametric resampling makes no assumptions concerning the distribution of the data. The algorithm for a nonparametric bootstrap is as follows 20:

1. Sample N observations randomly with replacement from the N cases to obtain a bootstrap data set.

2. Calculate the bootstrap version of IBMD.

3. Repeat steps 1 and 2 a B times to obtain an estimate of the bootstrap distribution.

For confidence intervals of 90–95 percent B should be between 1000 and 2000 21,22. In the results the confidence intervals were calculated with B equal to 1000.

### Software for IBMD assessment

#### Website

We have developed a website to assist with the calculation of IBMD and respective 95% confidence intervals 23. This site additionally includes computation of the intraclass correlation coefficient (ICC). Lin’s concordance correlation coefficient (CCC) and limits of agreement can also be measured when considering only two observations per subject. The website contains a description of these methods.

#### PAIRSetc software

PAIRSetc 24,25, a software that compares matched observations, provide several agreement measures, among them the ICC, the CCC and the 95% limits of agreement. This software is constantly updated with new measures introduced on scientific literature, in fact, a coefficient of individual equivalence to measure agreement, based on replicated readings proposed in 2011 by Pan et al. 26,27and IBMD, published in 2010, were already include.

### Examples

Two examples (one with real data and the other with hypothetical data) were employed to illustrate the utility of the IBMD in comparing the degree of disagreement.

A gymnast’s performance is evaluated by a jury according to rulebooks, which include a combination of the difficulty level, execution and artistry. Let us suppose that a new rulebook has been recently proposed and subsequently criticized. Some gymnasts and media argue that disagreement between the jury members in evaluating the gymnastics performance with the new scoring system is higher than that with the old scoring system, and therefore oppose its use. To better understand this claim, consider a random sample of eight judges evaluating a random sample of 20 gymnasts with the old rulebook, and a different random sample of 20 gymnasts with the new rulebook. In this case, each of the 40 gymnasts presented only one performance based on pre-defined compulsory exercises, and all eight judges simultaneously viewed the same performances and rated each gymnast independently, while blinded to their previous medals and performances. Both scoring systems ranged from 0 to 10. The results are presented in Table 1.

**Table 1:** Performance of 40 gymnasts, 20 evaluated by eight judges using the old rulebook and 20 by the same judges using the new rulebook

**ID gymnast**	**Rulebook**	**Jude 1**	**Jude 2**	**Jude 3**	**Jude 4**	**Jude 5**	**Jude 6**	**Jude 7**	**Jude 8**
1	Old	7.10	7.20	7.00	7.70	7.10	7.10	7.00	7.30
2	Old	9.30	9.70	8.90	9.60	8.60	9.50	9.60	9.70
3	Old	8.90	8.80	8.10	9.30	8.50	8.10	7.60	8.70
4	Old	8.00	8.10	7.30	8.70	7.50	8.70	7.40	9.50
5	Old	9.10	9.00	8.20	9.00	8.20	9.50	7.80	8.00
6	Old	9.10	9.20	8.30	9.10	7.90	8.90	9.00	9.20
7	Old	8.90	9.00	7.70	9.00	8.00	9.40	8.00	7.70
8	Old	8.30	8.70	8.10	8.90	7.80	9.20	7.80	9.30
9	Old	9.30	9.40	8.20	9.40	8.80	9.30	9.20	9.80
10	Old	9.40	9.80	9.40	9.70	9.10	10.00	9.30	9.60
11	Old	7.70	8.70	7.60	9.00	7.70	8.50	7.70	7.70
12	Old	9.20	9.70	8.50	9.60	8.60	9.90	9.70	7.40
13	Old	7.40	7.30	7.10	7.90	7.10	7.40	7.00	7.50
14	Old	8.40	8.90	7.40	8.60	7.80	8.10	7.40	8.90
15	Old	7.40	7.60	7.10	8.10	7.20	7.60	7.10	8.80
16	Old	9.80	9.90	9.20	9.80	9.30	10.00	9.40	9.60
17	Old	9.60	9.60	9.50	9.80	9.10	9.90	9.40	9.90
18	Old	9.60	9.80	9.50	9.80	8.80	9.90	9.80	9.20
19	Old	8.50	9.20	7.80	9.30	7.90	9.00	7.70	9.70
20	Old	7.10	9.50	8.80	9.40	8.50	9.60	7.90	8.50
21	New	6.50	8.20	6.60	9.80	7.50	7.80	6.10	5.10
22	New	7.00	9.70	7.60	9.60	8.30	6.90	6.70	8.60
23	New	7.50	8.60	6.60	7.80	9.50	8.10	6.20	7.60
24	New	8.50	9.00	8.10	7.00	8.30	9.40	6.70	8.00
25	New	9.70	8.10	7.50	6.80	7.70	8.60	8.30	7.40
26	New	8.00	9.10	7.40	9.30	8.30	9.70	6.00	9.90
27	New	7.80	9.70	7.00	9.70	8.70	10.00	9.60	9.50
28	New	9.30	7.90	8.20	7.80	6.30	7.40	6.10	7.20
29	New	7.10	9.80	8.10	9.50	6.30	9.40	8.90	6.50
30	New	8.90	9.30	7.90	6.80	8.20	9.10	7.90	6.80
31	New	9.30	9.80	8.80	6.60	8.50	9.80	7.40	9.90
32	New	7.90	8.20	6.70	9.40	7.60	6.10	7.40	7.10
33	New	7.60	8.50	6.40	8.50	9.20	7.80	6.20	9.40
34	New	8.60	8.90	6.50	9.00	7.70	9.10	6.50	7.10
35	New	8.80	7.20	8.80	9.30	8.40	9.30	6.90	8.60
36	New	8.40	9.30	7.50	8.70	7.90	9.60	7.90	7.90
37	New	7.50	8.00	7.20	8.40	7.40	7.20	9.10	9.20
38	New	9.70	9.80	9.50	9.80	9.00	9.90	9.40	9.60
39	New	8.50	9.20	8.70	9.30	7.00	9.70	8.30	8.00
40	New	7.30	8.70	7.20	8.10	7.30	7.30	7.10	7.20

Visual analysis of the maternal heart rate during the last hour of labor can be more difficult than that during the first hour. We believe that this is a consequence of the deteriorated quality of signal and increasing irregularity of the heart rate (due to maternal stress). Accordingly, we tested this hypothesis by examining whether in visual analysis of cardiotocograms, observer disagreement in fetal heart rate baseline estimation in the first hour of labor is lower than that in the last hour of labor when different observers assess printed one-hour cardiotocography tracings. To answer this question, we evaluated the disagreement in maternal heart rate baseline estimation during the last and first hour of labor by three independent observers.

Specifically, the heart rates of 13 mothers were acquired, as secondary data collected in Nélio Mendonça Hospital, Funchal for another study, during the initial and last hour of labor, and printed. Three experienced obstetricians were asked to independently estimate the baseline of the 26 one-hour segments. Results are presented in Table 2. The study procedure was approved by the local Research Ethics Committees and followed the Helsinki declaration. All women who participate in the study gave informed consent to participate.

**Table 2:** Estimation of baseline (bpm) in 26 segments of 13 traces (13 segments corresponding to the initial hour of labor and 13 to the final hour of labor) by three obstetricians

**Mother ID**	**Segment**	**Obstetrician 1**	**Obstetrician 2**	**Obstetrician 3**
1	Initial hour	80	80	80
2	Initial hour	65	66	70
3	Initial hour	65	66	70
4	Initial hour	63	67	65
5	Initial hour	82	83	85
6	Initial hour	75	76	75
7	Initial hour	80	81	85
8	Initial hour	84	85	80
9	Initial hour	100	102	105
10	Initial hour	82	82	80
11	Initial hour	67	65	70
12	Initial hour	75	74	87
13	Initial hour	70	70	70
1	Last hour	78	75	75
2	Last hour	90	90	100
3	Last hour	70	67	70
4	Last hour	70	65	65
5	Last hour	87	87	90
6	Last hour	72	73	75
7	Last hour	75	75	75
8	Last hour	100	98	100
9	Last hour	110	108	110
10	Last hour	103	103	100
11	Last hour	80	80	100
12	Last hour	98	100	100
13	Last hour	70	70	65

## Results

### Hypothetical data example

Using IBMD in the gymnast’s evaluation, we can compare observer disagreement and the respective confidence interval (CI) associated with each score system.

The disagreement among judges was assessed as IBMD = 0.090 (95%CI = [0.077;0.104]) considering the old rulebook and IBMD = 0.174 (95%CI = [0.154;0.192]) with new rulebook. Recalling that the value 0 of the IBMD means no disagreement (perfect agreement), these confidence intervals clearly indicate significantly higher observer disagreement in performance evaluation using the new scoring system, compared with the old system.

### Real data example

The disagreement among obstetricians in baseline estimation, considering the initial hour of labor, was IBMD = 0.048 (95%CI = [0.036;0.071]), and during the last hour of labor, IBMD = 0.048 (95%CI = [0.027;0.075]). The results indicate no significant differences in the degree of disagreement among observers between the initial and last hour of labor.

## Discussion

While comparison of the degree of observer disagreement is often required in clinical and epidemiologic studies, the statistical strategies for comparative analyses are not straightforward.

Intraclass correlation coefficient is several times used in this context, however sometimes without careful in choosing the correct form. Even when the correct form of ICC is used to assess agreement, its dependence on variance does not always allow the comparability of populations. Other approaches to assess observer agreement have been proposed 28,29,30,31,32,33, but comparative analysis across populations is still difficult to achieve. The recently proposed IBMD is a useful tool to compare the degree of disagreement in non-negative ratio scales 18, and its proposed generalization allowing several observers overcomes an important limitation of this measure in this type of analysis where more than two observers are required.

## Conclusions

IBMD generalization provides a useful tool to compare the degree of observer disagreement among different methods, populations or circumstances and allows evaluation of data by different numbers of observers for different cases, an important feature in real situations where some data are often missing.

The free software and available website to compute generalized IBMD and respective confidence intervals facilitates the broad application of this statistical strategy.

## Competing interests

There are any non-financial competing interests (political, personal, religious, ideological, academic, intellectual, commercial or any other) to declare in relation to this manuscript.

## Authors’ contributions

TH, LA and CCS have made substantial contributions to article conception and design. They also have been involved the analysis and interpretation of data and they draft the manuscript. JB have been involved in article conception and he revised the manuscript critically for important intellectual content. MM and DS were responsible for the creation of the software and also were involved in the data analysis. All authors read and approved the final manuscript.

## Pre-publication history

The pre-publication history for this paper can be accessed here:

http://www.biomedcentral.com/1471-2288/13/47/prepub
